# Extended PRF: Impact of Heat on Gene Expression in Gingival Fibroblasts

**DOI:** 10.3390/ijms26189120

**Published:** 2025-09-18

**Authors:** Xiaoyu Huang, Layla Panahipour, Dorna Rassi Faghihi, Richard J. Miron, Reinhard Gruber

**Affiliations:** 1 Department of Oral Biology, University Clinic of Dentistry, Medical University of Vienna, 1090 Vienna, Austria; huangxiaoyu_smu@163.com (X.H.); layla.panahipour@meduniwien.ac.at (L.P.); n11705843@students.meduniwien.ac.at (D.R.F.); 2 Department of Prosthodontics, The Affiliated Stomatological Hospital of Southwest Medical University, Luzhou 646000, China; 3 Department of Periodontology, School of Dental Medicine, University of Bern, 3010 Bern, Switzerland; richard.miron@unibe.ch

**Keywords:** albumin, fibrin, platelet-rich fibrin, platelet-poor plasma, chemokine, TGF-β

## Abstract

Extended platelet-rich fibrin (e-PRF) combines the prolonged resorption properties of heat-coagulated platelet-poor plasma (PPP), becoming an albumin gel (Alb-gel) that is mixed back with the respective native cell-rich buffy coat layer (BC), i.e., concentrated PRF (C-PRF). E-PRF or Alb-PRF is utilized as a barrier membrane in various clinical applications, such as guided tissue regeneration. The heating of PPP might lower its biological activity, but testing this hypothesis is necessary. To this end, we exposed gingival fibroblasts to the lysates of regular PPP, heated PPP (hPPP), and BC, followed by bulk RNA sequencing. Gingival fibroblasts responded to PPP lysates with a total of 153 up- and 71 down-regulated genes when considering a minimum 3.0-fold log2 expression change and a significance level 2.0 log-10. In sharp contrast, the response to hPPP was characterized by only five up-regulated and five down-regulated genes, clearly indicating that heating almost completely abolished the biological activity of PPP. As expected, BC was more potent than PPP and broadened the spectrum of regulated genes. RT-PCR and immunoassays confirmed the heat sensitivity of PPP as exemplified by IL11 and other genes. Moreover, PPP, but not hPPP, drives the phosphorylation of p65, representing NF-κB signaling. Taken together, these findings extend previous observations that PPP causes a robust response in gingival fibroblasts and also strengthen the hypothesis that this response is heat-sensitive. These operations support the clinical concept of e-PRF by mixing back the heated inactive PPP with the bioactive buffy coat C-PRF layer.

## 1. Introduction

Oral tissues can undergo permanent rejuvenation during homeostasis [1]. Also, the recovery potential is enormous, allowing for empty sockets to heal spontaneously in an unsterile and mechanically unstable environment [1]. However, tooth loss gradually leads to bone atrophy, and thus a catabolic modeling of the original anatomical structures [2,3,4]. This is where reconstructive or regenerative dentistry becomes relevant, introducing classical therapeutic strategies, where, for instance, the augmented defect site is shielded away from the surrounding tissues by a barrier, usually a collagen or non-resorbable membrane [5,6,7,8,9]. The principle of guided bone and tissue regeneration, however, does not dictate which membrane to use as compulsory; therefore, strategies to extend the current portfolio of options for membranes have been developed. One strategy utilizes autologous membranes, such as those prepared from the patient’s blood. This strategy is a variation of the fundamental concept of using fractionated blood, allowing for spontaneous coagulation, which is based on the concept of platelet-rich fibrin, known as PRF [10,11,12,13,14].

To prepare PRF, blood is fractionated into liquid plasma and its cellular components. Depending on the relative centrifugation forces, the angle of the tubes during centrifugation, and the time, the protocols can be modified [15]. When blood is subjected to high-speed centrifugation, which is 2000× *g* or more for approximately 8 min, it separates into platelet-poor plasma (PPP) and the buffy coat layer; the latter accumulates at the interface with the heavy red blood cells at the bottom of the tubes [15]. The PPP is poor but not entirely devoid of platelets; platelets, when activated during processing, stick to the fibrin and fibronectin and thus become a vital part of PPP [16,17]. Fractionated PPP can now be left for spontaneous coagulation, later followed by fibrinolysis, requiring the activation of intrinsic but heat-sensitive plasminogen [18,19,20]. Recently, an alternative approach has been proposed to prolong resorption properties by inhibiting fibrinolysis. Heating PPP (hPPP) at 75 °C for 10 min causes the coagulation of temperature-sensitive albumin; however, under these conditions, plasminogen is also denatured, independent of the coagulation cascade [21]. Since heating not only denatures structural proteins but also inactivates intrinsic fibrinolytic enzymes [19], heated PPP is resistant to fibrinolysis and may thus hold clinically relevant membrane properties [22]. However, the question arises about whether heating affects the biological properties of spontaneously coagulated PPP.

The biological properties of PPP, similar to PRF, are described by the growth factors and other molecules stored in the fibrin-rich matrix [23,24]. For instance, growth factors such as TGF-β can be measured via an immunoassay [25,26,27], but more convincingly, by causing a strong response in a bioassay. This response is exemplified by the increased expression of IL11 in gingival fibroblasts exposed to PPP lysates, an effect that significantly diminished upon heating PPP at 75 °C for 10 min [28]. Thus, the TGF-β activity of PPP is heat-sensitive [28], which is why the hPPP, the albumin gel (Alb-gel), is mixed back with the native cell-rich buffy coat layer (BC), the concentrated PRF (C-PRF), to regain bioactive TGF-β [21]. Previous bioassays, however, only entail a small gene panel, and do not represent the overall activity of PPP and the effect of its heating [28]. Considering the increasing clinical relevance of the local application of e-PRF with its hPPP/Alb-gel and BC/C-PRF components [29], there is a demand to better understand how heating affects the biological properties of PPP.

The present research, therefore, fuses and extends two significant previous observations, namely that hPPP cannot increase the expression of IL11 in gingival fibroblast [28] and that, based on RNAseq, PRF lysates cause a robust increase in chemokines and cytokines, such as CXCL2, CXCL5, CXCL6, and IL33 [30]. Hence, new questions arise: First, how potent is PPP, being almost devoid of cellular components, to change the transcriptome of gingival fibroblasts? Second, to what extent is the intrinsic activity of PPP heat sensitive? Our bioassay follows the previous strategy, where we have identified the heat sensitivity of PPP based on IL11 expression in fibroblasts, and the RNAseq approach with PRF lysates and serum. Our research revealed a strong cellular response to PPP, and upon heating PPP, there was almost a complete lack of transcriptomic changes in gingival fibroblasts.

## 2. Results

### 2.1. Principal Component Analysis and Heat Map of Gene Expression by PPP, hPPP, and BC

**Figure 1:**
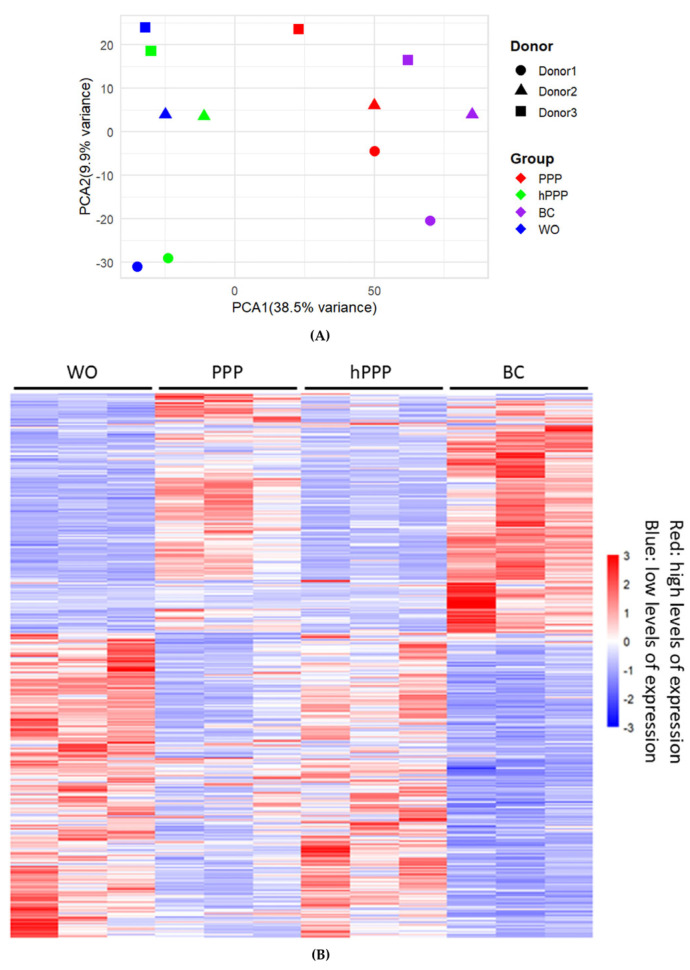
(**A**) Principal component analysis (PCA) reveals donor homogeneity within treatment groups and heterogeneity among treatments for gingival fibroblasts treated with 30% PPP (red), hPPP (green), or BC (purple). Untreated cells (WO) are shown in blue. (**B**) Corresponding heat map analysis of differential expressed genes (adjusted *p* < 0.05, log2 fold change ≥ 1 or ≤−1) are shown; rows represent genes, columns represent treatment groups. Red indicates high expression; blue indicates low expression.

To investigate the effect of PPP, hPPP, and BC related to transcriptional signature on human gingival fibroblasts, we conducted the single-cell RNA sequencing of gingival fibroblasts pooled from three donors and exposed to 30% PPP, hPPP, and BC from three independent donors overnight. Principal component analysis (PCA) revealed good reproducibility within the PPP, hPPP, and BC donors, and the different treatments can be separated into two dimensions. There was, however, a variation coming from the three independent cell donors in PC2. The PC1 shift caused by PPP and even more with BC, however, was consistent among the three donors (Figure 1A). Thus, the variance is mainly at the level of the fibroblasts but not at the level of plasma fractions. Consistently, the heat map analysis revealed a variation in the basal expression pattern among the three fibroblast donors. Transcriptional heterogeneity between gingival fibroblasts exposed to PPP and BC was evident in the heat map (Figure 1B). Nevertheless, hPPP clearly resembles the transcriptional pattern of the untreated cells, supporting the corresponding minor shift in the PCA. Thus, only a minor gene expression changes can be expected with hPPP.

### 2.2. Volcano Analysis of Gene Expression Changes by PPP, hPPP, and BC

**Figure 2:**
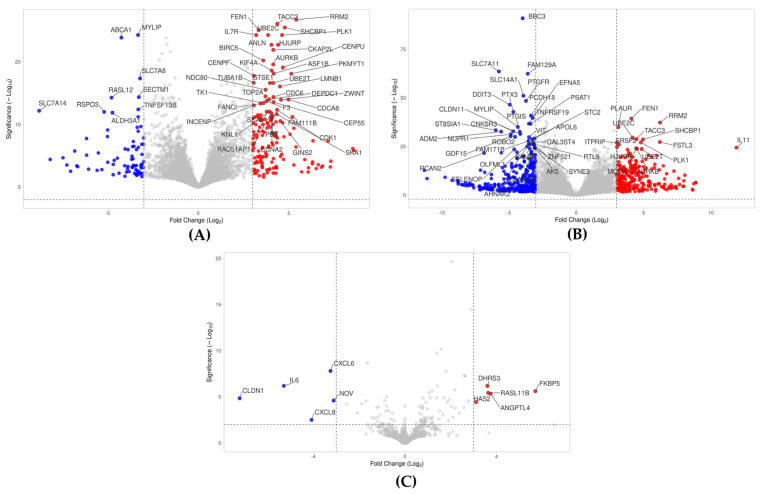
(**A**) Volcano plot analysis of differentially expressed genes in gingival fibroblasts treated with PPP, with up-regulated (red) and down-regulated (blue) genes. The annotated dots revealed 50 hits based on Manhattan distance (out of 224 genes). (**B**) Gingival fibroblasts treated with BC were limited to 50 hits based on Manhattan distance (out of 563 genes), and (**C**) hPPP only showed 10 hits that reach the threshold level. The selection of this stringent threshold was possible as gingival fibroblasts show a high magnitude of gene expression changes caused by PPP and BC, also underlying the loss of activity upon heating PPP.

Volcano plot analysis identified 153 up-regulated and 71 down-regulated genes in gingival fibroblasts treated with PPP lysates (|log_2_FC| ≥ 3; −log_10_(*p*) ≥ 2.0) (Figure 2A, Supplementary Table S1). Consistent with the same premise as PPP, it was demonstrated that 251 and 312 genes were up- and down-regulated by BC, respectively (Figure 2B, Supplementary Table S2). As already anticipated in the PCA and heat map analysis, the volcano analysis of hPPP revealed only five up- and five down-regulated genes under these conditions (Figure 2C, Supplementary Table S3). The stringent threshold covered a high magnitude of gene expression changes caused by PPP and BC, and the substantial activity loss upon heating PPP. Thus, heating significantly diminished the transcriptomic impact of PPP, and the response of gingival fibroblasts was purposefully stronger with lysates from BC compared to those obtained from PPP.

### 2.3. Venn and UpSet Analyses of Genes Regulated by PPP, hPPP, and BC

**Figure 3:**
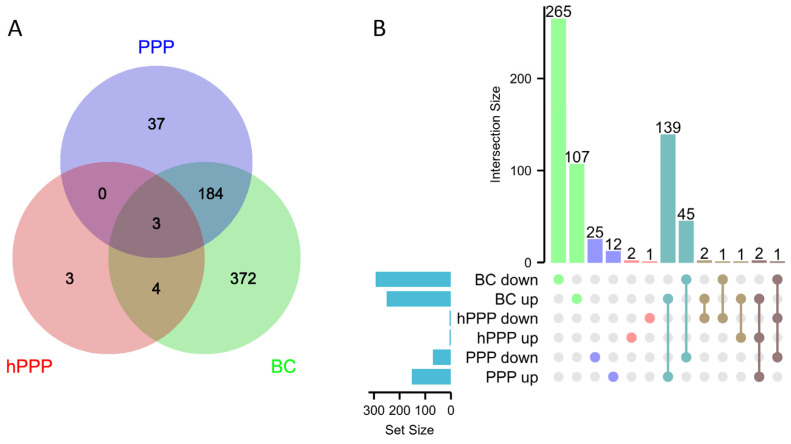
(**A**) A Venn diagram shows the number of genes regulated by PPP, hPPP, and BC, respectively, or interactively. (**B**) UpSet plot analysis of differentially expressed genes (DEGs) in gingival fibroblasts. The bar chart above represents the number of genes contained in each type of group. The bar chart at the bottom left represents the number of events included in each type of event. The dotted line at the bottom right shows the types of events contained in the group.

Then, we conducted a Venn and UpSet analyses to display the differences and interactions in gene expression with the presence of PPP, hPPP, and BC. The Venn diagram (Figure 3A) showed that BC uniquely modulated 372 genes, substantially exceeding PPP, which had 37 genes, and only 3 genes, including IL6, with hPPP. Moreover, PPP and BC co-regulated 184 genes. Notably, only HAS2, FKBP5, and CLDN1 overlapped with PPP, hPPP, and BC. Showing the differentially expressed genes (DEGs) in gingival fibroblasts, the UpSet plot displayed that BC exerted a broad influence (563 DEGs), predominantly down-regulatory (265 genes). UpSet analysis further showed that among the genes exclusively regulated by BC, a majority are down-regulated (265 vs. 107), which is also the case with PPP (25 vs. 12). In contrast, the genes were common to both BC and PPP, most of which are up-regulated rather than down-regulated (139 vs. 45, respectively).

Two questions arise from this analysis: (i) How does hPPP regulate the 10 genes? What is the heat-stable molecular trigger, and does IL6 even require heating of PPP to be activated? (ii) There is a substantial overlap of BC and PPP, mainly for up-regulated genes, but also, particularly, an independent change in the transcriptome that needs special attention for BC as it may explain what BC can add to the overlay system—here, the e-PRF—as covered in more detail in the following analysis.

### 2.4. G:Profiler Analysis of Gene Expression Changes by PPP, hPP, P and BC

**Figure 4:**
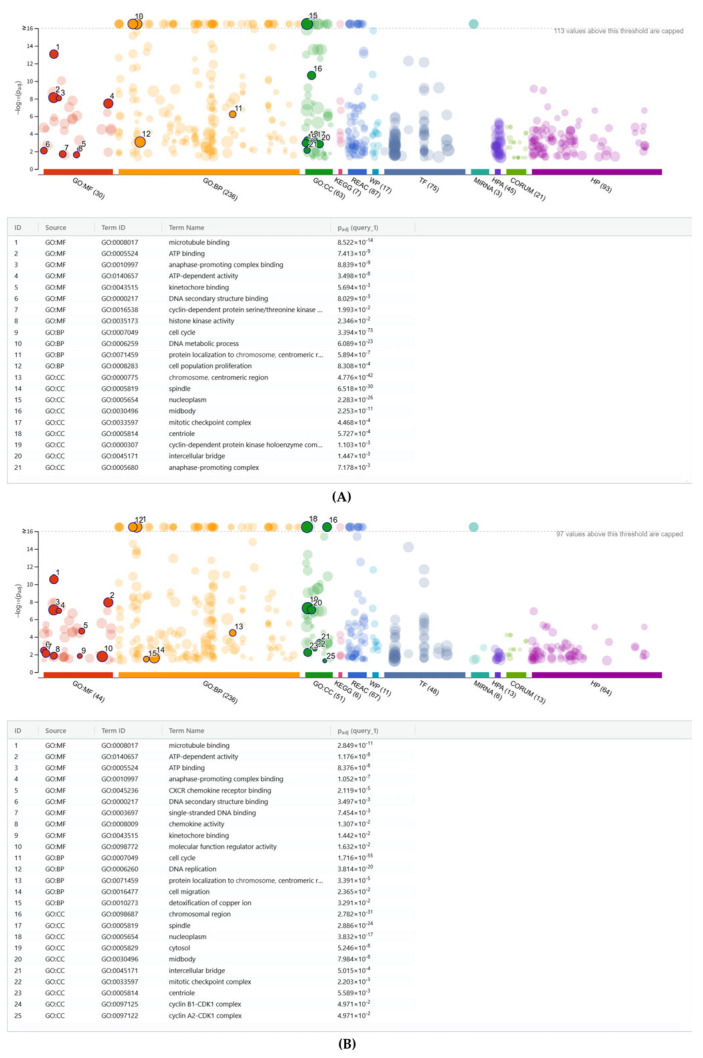
Functional enrichment analysis of up-genes regulated by (**A**) PPP and (**B**) BC. G: Profiler results are displayed as a Manhattan plot, where the x-axis organizes enriched functional terms by source database (color-coded). The y-axis shows FDR-adjusted *p*-values transformed as-log_10_. Gene Ontology (GO) annotations encompass three primary categories: Biological Process (BP), Molecular Function (MF), and Cellular Component (CC) terms.

To understand the potential biological impact of the signature changes, we further performed over-representation analysis (ORA) or gene set enrichment analysis. A summary of the analysis is provided in Supplementary Tables S4–S9. As shown in Figure 4A and Supplementary Table S4, the gene ontology analysis of all 153 genes up-regulated by PPP revealed gene ontology (GO) categories for biological processes (BP), molecular functions (MF), and cellular components (CC). Almost in unison, this enrichment analysis suggests that PPP regulates the genes involved in cell mitosis and related mechanisms, for instance, GO: BP cell population proliferation GO:0008283 with 35:2009 genes. Among the down-regulated genes is GO: MF cytokine activity GO:0005125 with SECTM1, TNFSF13B, CCL11, TNFSF10, TGFB3, IL1RN, and GDF10, suggesting a moderate reduction in basal cytokine signaling, but in a non-challenged situation (Supplementary Figure S1 and Table S5). Importantly, with the heating of PPP, no enrichment of upregulated gene was observed. GO: MF represents the gene ontology of down-regulated genes, including CXCR chemokine receptor binding (GO:0045236, 2:18) and cytokine activity (GO:0005125, 3:239), which encompasses CXCL6, CXCL8, and IL6 individually (Supplementary Figures S3 and S4 and Tables S10 and S11).

With BC representing a lysate containing platelets, leucocytes, and plasma components, there is a more complex picture than as observed with PPP. There was an up-regulated gene enrichment by BC in both GO: MF chemokine activity (GO:0008009) and GO: MF CXCR chemokine receptor binding (GO:0045236), sharing the same genes: CXCL1, CXCL3, CXCL5, CXCL6, CXCL8, and PF4V1 from 6:50 genes and 6:18 genes, respectively. Thus, BC, but not PPP, provokes chemokine expression in gingival fibroblasts. Moreover, with 47:2120 genes, GO: MF molecular function regulator activity (GO:0098772) further identifies additional paracrine molecules such as IL11, IL33, STC1, BMP2, AREG, etc. Furthermore, also notable is GO: BP (GO:0010273), which is indicated by 4:14 genes, including MT1X, MT2A, MT1A, and MT1M, related to the detoxification of copper ions (Figure 4B, Supplementary Table S6). As an illustration for the down-regulated genes by BC, GO: MF signaling receptor regulator activity GO:0030545 with 22:554 genes, comprising a series of genes, including GDF5, GDF15, BMP4, FGF13, FGF18, IL34, WNT2B, and CCL11 showing paracrine factors, is also diminished by BC (Supplementary Figure S2 and Table S7).

### 2.5. PPP, Buffy Coat, and E-PRF Lysates, but Not Heated PPP, Enhance Gene Expression

**Figure 5:**
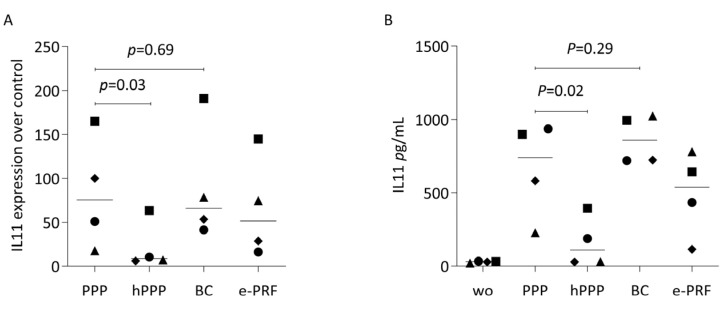
PPP, buffy coat, and e-PRF induced a substantial increase of IL11 in gingival fibroblasts, showing expression as x-fold increases compared with untreated cells. (**A**) Real-time PCR analysis of IL11. The expression level of the untreated cells was set to 1 for calibration. (**B**) Quantification of IL11 levels in the supernatant by immunoassay. Statistical analysis was based on the ratio-paired *t*-test. Wo stood for the untreated cells. N = 4, and different symbol shapes indicate independent experiments. Significance was set at *p* < 0.05.

**Figure 6:**
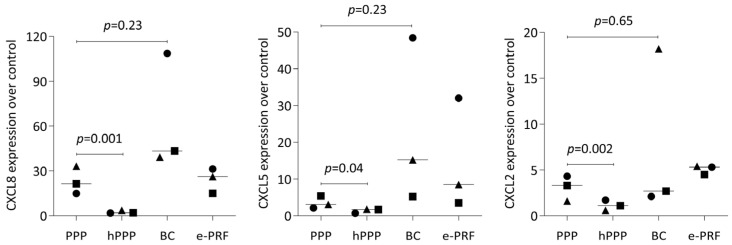
PPP, buffy coat, and e-PRF, but not heated PPP, induced a substantial increase of CXCL8, CXCL5, and CXCL2 showing expression as x-fold increases. Statistical analysis was based on the ratio-paired *t*-test. N = 3, and different symbol shapes indicate independent experiments. Significance was set at *p* < 0.05.

To confirm the selected findings from bulk RNA sequencing, we analyzed the effects of lysates prepared from PPP, heated PPP, BC, and e-PRF on the expression changes of IL-11 in human gingival fibroblasts. IL11 expression changes are caused by the TGF-beta activity of plasma components [28]. We observed the expected increase in IL11 expression by real-time PCR when the fibroblasts were exposed to the lysates of PPP, buffy coat and the combination of e-PRF, but the increase was significantly less evident with hPPP alone (Figure 5A). Moreover, we measured the protein levels of IL11 in the supernatant of the gingival fibroblasts, obtaining a similar pattern. Some of the TGF-beta activity causing the IL11 increase remains upon heating PPP at least in two out of the four experiments. (Figure 5B). We also found that PPP increases the chemokines CXCL8, CXCL5, CXCL2, in gingival fibroblasts, however, not with heated PPP (Figure 6).

### 2.6. Lysates of PPP, but Not Heated PPP, Induced the Phosphorylation of p65

**Figure 7:**
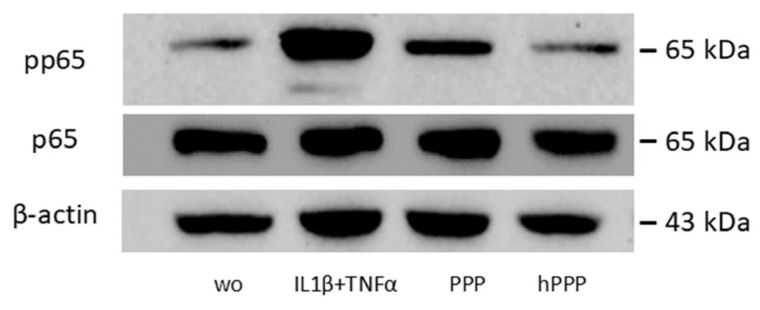
Lysates of PPP but not heated PPP provoked phosphorylation of p65. Gingival fibroblasts were exposed for 1 h to IL1β + TNFα, and 30% of the lysates prepared from PPP, heated PPP (Alb-gel) (75 °C for 10 min), respectively. Western blot showed a substantial increase in the basal p65 phosphorylation signal by lysates prepared from PPP but not when cells were exposed to lysates of heated PPP.

To investigate the potential role of PPP and heat-activated PPP in the NF-κB signaling pathway, we performed Western blot analysis. Knowing that phosphorylation of p65 is a prerequisite for its nuclear translocation, it was demonstrated that the PPP lysates activated the increased phosphorylation of p65, whereas heated PPP had no effect (Figure 7). The NF-κB analysis was inspired by the PCR data that not only BC lysates but also PPP moderately increases chemokine expression in fibroblasts, thus supporting the hypothesis that PPP can induce moderate NF-κB signaling, and more importantly, that heating PPP removes this capacity.

## 3. Discussion

This research was prompted by the recently established heated PPP (Alb-gel) mixed with C-PRF, which is the BC, to serve as a long-term stable autologous and injectable matrix for regenerative medicine [21,31]. While there is accumulating evidence for the complex cellular response to PPP, BC, and its lysates, little is still known about the impact of heating. First attempts showed that PPP and BC, but significantly less heated PPP, induced a substantial increase in TGF-β target genes, including IL11, NOX4 [28], and decreased a forced inflammatory response in hematopoietic cells [32], or neutralized H_2_O_2_ cytotoxicity in fibroblasts [33]. These original findings may sound surprising as PPP is considered poor in cellular components, at least from a clinical perspective. Moreover, even though bioassays highlight the activity intrinsic to PPP, all bioassays were limited to a restricted selection of potential target genes. Thus, it cannot be ruled out that heated PPP maintains at least part of its original activity. It is possible that a heat-stable fraction of PPP may change the genetic signature of potential target cells. The present research is, therefore, important for two primary reasons. First, we present the intense and complex genomic response of gingival cells being exposed to PPP and compare this to the strong activity of BC lysates. Second, we demonstrate that heated PPP almost lost its entire activity, at least with respect to driving gene expression changes in gingival fibroblasts.

If we relate our findings to those of others, our RNA sequencing data on PPP are in agreement with the strong cellular response we recently reported for gingival fibroblasts exposed to PRF membrane lysates and PRF serum, the latter being the liquid fraction of the original PRF [30]. The present research focused on the impact of PPP and its heating, also implementing RNA sequencing as a screening tool. This analysis revealed that it is primarily the genes involved in cell division that are significantly up-regulated by PPP lysates, thereby supporting previous findings obtained with the release of activated platelets, causing a robust increase in cell proliferation [34]. Also, in agreement with the previous findings [28,32,33] are observations that heated PPP was significantly less active compared to unheated PPP, at least under the conditions for preparing e-PRF [21]. Impressively, only five up- and five down-regulated genes were significantly regulated by heated PPP. Obviously, almost the entire activity of PPP, which caused 153 and 71 genes to be up- and down-regulated in gingival fibroblasts, was diminished by heating. Thus, hPPP was no longer mitogenic.

Since BC was not heated when preparing e-PRF and, in contrast to PPP, also rich in cellular components, we expected a massive response when cultured with gingival fibroblasts. Indeed, the lysates of BC additionally induced the expression of 372 genes that were not changed by PPP. Thus, apart from the mitogenic activity indicated by the mitosis genes already identified by PPP, BC lysates hold the ability to significantly drive chemokine expression in the gingival fibroblasts, indicated by CXCL6, CXCL5, CXCL8, CXCL1, and CXCL3, similar to what we have observed with PRF lysates and serum [30]. Hypothetically, because PPP is poor in cells and BC is the opposite, it is the cellular components of fractionated blood, including platelets and leucocytes, that cause fibroblasts to significantly express chemokines. One possible explanation is that the platelet release CD40 ligand [35] and gingival fibroblasts expressing the respective CD40 receptor, upon activation, drive cytokine [36] but presumably also chemokine production, as reported for cervical carcinoma cells [37]. This is an exciting research question that originates from the present observations. Also unique to BC lysates were the increased expressions of MT1X, MT2A, MT1A, and MT1M, all genes related to heavy metal detoxification and a potent antioxidant [38,39]. This is potentially caused by the metal ions that are released into a lysate upon sonification and freeze/thawing [40,41]. Thus, care should be taken when interpreting the findings, as BC lysates consist of a mixture of necrotic cells, rather than the intact cell population. Thus, the components of the cytoplasm may provoke a cell response that is perhaps more representative of a necrotic cell lysate, as we have previously reported for other cell types [42]. Thus, from a clinical perspective, the BC data should be interpreted carefully.

The clinical relevance of the findings leads us to speculate. Firstly, heated PPP provided a long-lasting autologous matrix, where the coagulated PPP maintained its original volume [43]. By mixing back the BC, at least some of heat-sensitive PPP activity was partially reversed [21]. Thus, e-PRF is not per se inactive, as the freshly prepared and non-heated BC brings back the activity to the heated PPP. However, e-PRF should not be compared to PRF, or sticky bone where PRF membranes form a conglomerate with bone particles and natural coagulated PPP [44], as it is not clear how the local cellular response is affected by heat-coagulated PPP, particularly because the fibrinolysis is diminished, and, therefore, also vascular invasion and the immigration of leucocytes. It can be speculated that the clinical indication, for instance, as a filler or transiently stable membrane, does not require the potent biological activity of PPP, but rather the stable maintenance and cell-shielding properties of the autologous denatured blood components of heated PPP.

The study has significant limitations, namely that our RNA-seq approach underlines the almost-negligible activity of heated PPP in our in vitro bioassay with fibroblasts. In vivo, however, the situation is more complex, and the question arises as to the impact of heated PPP on cells of the leukocyte lineage, for instance, macrophages. Future research, at least in the in vitro setting, should focus on how heating PPP affects the response of macrophages, as human peripheral blood mononucleated cells are capable of responding to PRF lysates in vitro, including the expression of chemokines [45]. It is now important to consider the RNA sequencing technology to decipher the response of macrophages to PPP and its heated version. Moreover, as the blood clot and particular PRF are supposed to support wound healing, most obviously in diabetic foot ulcers and vein leg ulcers [46], the question arises on the impact of PPP and therefore also heated PPP on epithelial target cells, not necessarily limited to the oral cavity, but also extending this question toward the field of dermatology and orthopedic medicine [47,48,49]. Perhaps another limitation of the present research is that we have not put our focus on the interpretation of the genetic signature changes. Nevertheless, BC is more complex than PPP which basically shows mitosis gene expression changes. This is not surprising, as BC lysates are rich in cellular fragments compared to the rather cell-poor PPP fraction. It is, thus, even more impressive the way in which PPP enhanced the expression change in gingival fibroblasts.

In conclusion, our research demonstrates the cellular response of oral fibroblasts exposed to lysates of PPP, which is less complex when compared to the lysates of BC, but still strong, and the surprisingly robust blocking of the overall biological activity of PPP by heating for 10 min at 75 °C. Clinically, these findings support the approach of mixing back BC with heated PPP, i.e., Alb-PRF, to regain at least part of its original activity while maintaining the expected low degradation or resorption of the final product, i.e., e-PRF.

## 4. Materials and Methods

### 4.1. Cell Culture

Approval for collecting human gingiva was obtained from the Ethics Committee of the Medical University of Vienna (EK NR 631/2007), and patients signed informed consent. Three different individuals of fibroblasts were prepared from explant cultures, and passaged less than 10 times. Gingival fibroblasts were expanded in Dulbecco’s modified Eagle’s medium (DMEM, Sigma-Aldrich, St. Louis, MO, USA) containing penicillin, streptomycin (Sigma Aldrich, St. Louis, MO, USA), and 10% fetal bovine serum (Bio&Sell GmbH, Nuremberg, Germany). Fibroblasts at 30,000 cells/cm^2^ were exposed to the lysates of unheated and heated PPP (Alb-gel), buffy coat (C-PRF/BC), and extended PRF (e-PRF) in a serum-free medium for 24 h at 37 °C, 5% CO_2_, and 95% humidity.

### 4.2. Preparation of PPP, Heated PPP, Buffy Coat, and E-PRF Lysate

Following approval by the ethics committee of the Medical University of Vienna (1644/2018), volunteers signed informed consent forms before blood drawing. Venous blood was collected from healthy donors, three females aged from 23 to 35 years, in plastic tubes (“No Additive”, Greiner Bio-One GmbH, Kremsmünster, Austria), and centrifuged at 2000× *g* for 8 min (swing-out rotor; Z306 Hermle, Universal Centrifuge, Wehingen, Germany). The uppermost 2 mL of PPP and the 1–2 mL of concentrated liquid PRF was collected and pooled, respectively. Thereafter, PPP and BC were put on the cooling device to extend the clotting time. To generate Alb-gel [21], PPP was immediately heated at 75 °C for 10 min (Eppendorf, Thermomixer F1.5, Hamburg, Germany) before being placed on the cooling device for at least 2 min. To generate e-PRF, 1 mL of cooled Alb-gel was transferred into a petri dish, then mixed thoroughly with 1 mL of cooled BC. For each 1 mL fraction of e-PRF, it was mixed with 1 mL of filtered serum-free media. All the fractions were subjected to dual freeze–thawing followed by sonication (Sonopuls 2000.2, Bandelin electronic, Berlin, Germany) for 30 s. After centrifugation (Eppendorf, Hamburg, Germany) at 15,000× *g* for 10 min, aliquots of lysates were stored at −20 °C for no longer than one month. Upon reaching the appropriate stimulation state, cells from independent donors were exposed to the thawed fractions also originating from independent donors as indicated above, thus being carried out as three entirely independent experiments.

### 4.3. RNA Sequencing

Total RNA was extracted using the GeneMATRIX Universal RNA Purification Kit (EUR_X_, Gdańsk, Poland) with rDNase set (Macherer-Nagel, Düren, Germany). Sequencing libraries from total RNA were prepared at the Core Facility Genomics, Medical University of Vienna, using the QuantSeq 3′ FWD protocol version 2 with unique dual indices (Lexogen GmbH, Vienna, Austria), following the low-input branch of the protocol. Seventeen PCR cycles were used for library prep, as determined by qPCR according to the library prep manual. Libraries were QC-checked on a Bioanalyzer 2100 (Agilent Technologies, Santa Clara, CA, USA) using a High Sensitivity DNA Kit for correct insert size and quantified using Qubit dsDNA HS Assay (Invitrogen, Waltham, MA, USA). Pooled libraries were sequenced on a P3 flowcell on a NextSeq2000 instrument (Illumina, San Diego, CA, USA) in 1 × 75 bp single-end sequencing mode. On average, 10 million reads were generated per sample. Reads in fastq format were generated using the Illumina bcl2fastq command line tool (v2.19.1.403) and the Lexogen idemux tool for optimal demultiplexing of long unique dual indices. Reads were trimmed and filtered using cutadapt version 2.8 to trim polyA tails, remove reads with N’s and trim bases with a quality of less than 30 from the 3′ ends of the reads. On average, 8.9 million reads were left after this procedure. Trimmed reads in fastq format were aligned to the human reference genome version GRCh38 with Gencode 29 annotations using STAR aligner [50] version 2.6.1a in 2-pass mode. STAR counted raw reads per gene. Differential gene expression was analyzed using DESeq2 [51] version 1.44.0 (DESeq2:lfcShrink method [52]). The sequence-based data described in this study are accessible through the Gene Expression Omnibus (GEO) public repository under the accession code GSE308088. These data were released on 25 September 2025.

### 4.4. Heat Map of Gene Expression Changes, Volcano Plot, Venn Diagram, and Gene Set Enrichment Analysis

A heat map was generated using R studio to visualize the differential gene expression between the experimental groups and control groups [53,54]. We used VolcaNoseR, a web-based tool, to generate a volcano plot. Genes meeting the criteria of |log_2_(fold change)| ≥ log_2_(2.5) and −log_10_(*p*-value) ≥ 2 were identified as up- or down-regulated, and selected for further analysis [55]. InteractiVenn and UpSet analyses, both web-based tools, were used to analyze gene sets through Venn diagrams and UpSet plots [56,57]. The g: Profiler was used as a functional enrichment analysis tool that integrates multiple databases, including Gene Ontology and KEGG [58].

### 4.5. Reverse-Transcription Quantitative Real-Time PCR (RT-qPCR) and Immunoassay

For RT-qPCR, total RNA was also prepared with the Gene MATRIX Universal RNA purification kit (EUR_X_, Gdańsk, Poland) with rDNase set (Machererey-nagel, Düren, Germany), followed by reverse transcription (LabQ, Labconsulting, Vienna, Austria) and polymerase chain reaction (LabQ, Labconsulting, Vienna, Austria) on a CFX ConnectTM Real-Time PCR Detection System (Bio–Rad Laboratories, Hercules, CA, USA). The primer sequences are listed in Table 1. The expression levels were calculated by normalizing to the housekeeping gene GAPDH using the ΔΔCt method. The immunoassay for human IL11 (DY218, R&D Systems, Minneapolis, MN, USA) was performed with the supernatant of gingival fibroblasts exposed to the lysates of PPP, heated PPP (Alb-gel), buffy coat (BC), and e-PRF.

### 4.6. Western Blot

Gingival fibroblasts seeded at 30,000 cells/cm^2^ were serum-starved overnight. Cells were exposed for 1 h to 10 ng/mL IL1β + 10 ng/mL TNFα, and 30% of lysates were prepared from PPP and heated PPP (Alb-gel), respectively. Protein extracts in SDS buffer containing protease and phosphatase inhibitors (complete ULTRA Tablets and PhosSTOP; Roche, Mannheim, Germany) were separated by SDS-PAGE and transferred onto PVDF membranes (Roche Diagnostics, Mannheim, Germany). The binding of rabbit p65 antibody (IgG, 1:1000, Cell Signaling Technology, #8242, Danvers, MA, USA) and rabbit phosphor-p65 antibody (IgG, 1:1000, Cell Signaling Technology, #3033, Danvers, MA, USA) was detected with HRP (anti-rabbit IgG, 1:10,000, Cell Signaling Technology, #CS-7074), respectively. Peroxidase was visualized with Clarity Western ECL Substrate (Bio-Rad Laboratories, Inc., Hercules, CA, USA) and signals detected with the ChemiDoc imaging system (Bio-Rad Laboratories, Inc., Hercules, CA, USA).

### 4.7. Statistical Analysis

All experiments were performed at least three times. Statistical analysis was performed with the ratio paired *t*-test. The results for the treatment groups were compared with those of the positive control group or the negative control group in the experiments. Analyses were performed using Prism v9 (GraphPad Software, La Jolla, CA, USA). Significance was set at *p* < 0.05.

## Figures and Tables

**Table 1:** Primer sequences.

Gene	Forward Sequence	Reverse Sequence
*CXCL2*	CCCATGGTTAAGAAAATCATCG	CTTCAGGAACAGCCACCAAT
*CXCL5*	AGCTGCGTTGCGTTTGTTTAC	TGGCGAACACTTGCAGATTAC
*CXCL8*	AACTTCTCCACAACCCTCTG	TTGGCAGCCTTCCTGATTTC
*IL11*	AAATAAGGCACAGATGCC	CCTTCCAAAGCCAGATC
*GAPDH*	AGCCACATCGCTCAGACAC	GCCCAATACGACCAAATCC

## Data Availability

The original raw sequencing data presented in the study are openly available in the NCBI Gene Expression Omnibus (GEO) repository under accession number GSE308088.

## Supplementary Materials

The following supporting information can be downloaded at: https://www.mdpi.com/article/10.3390/ijms26189120/s1.
