# Supplementary figures and images for: Extended PRF: Impact of Heat on Gene Expression in Gingival Fibroblasts
Source: Int J Mol Sci. 2025 Sep 18;26(18):9120. doi: 10.3390/ijms26189120 (PMC12470302; doi:10.3390/ijms26189120)

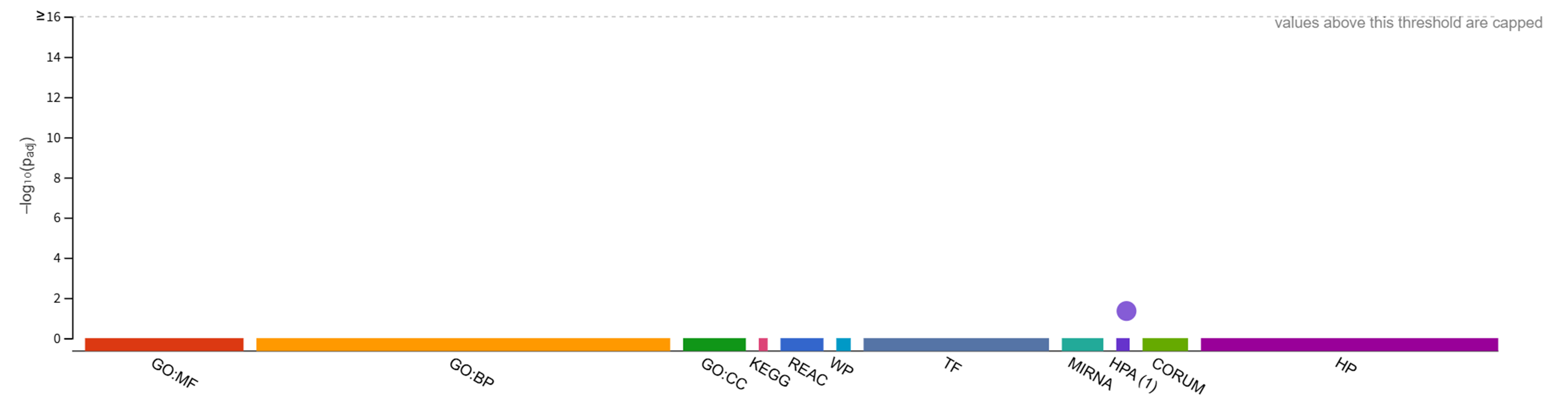

Supplement: Supplementary file 1 [file ijms-26-09120-s001.zip › ijms-3780438-Supplementary/Figure S3 hPPP upregulated.tif]
